# A Multicenter Retrospective Study of Elective Neck Dissection for T1-2N0M0 Tongue Squamous Cell Carcinoma: Analysis Using Propensity Score-Matching

**DOI:** 10.1245/s10434-018-07089-7

**Published:** 2018-12-04

**Authors:** Mitsunobu Otsuru, Yoshihide Ota, Souichi Yanamoto, Masaya Okura, Masahiro Umeda, Tadaaki Kirita, Hiroshi Kurita, Michihiro Ueda, Takahide Komori, Nobuhiro Yamakawa, Takahiro Kamata, Takumi Hasegawa, Takahiko Shibahara, Youichi Ohiro, Yoshihiro Yamashita, Kazuma Noguchi, Tadahide Noguchi, Kazunari Karakida, Hiroyuki Naito, Tomonao Aikawa, Tetsuro Yamashita, Daijiro Kabata, Ayumi Shintani

**Affiliations:** 1 Division of Surgery, Department of Oral and Maxillofacial Surgery, Tokai University School of Medicine, Isehara, Kanagawa Japan; 2 Unit of Translational Medicine, Department of Clinical Oral Oncology, Graduate School of Biomedical Sciences, Nagasaki University, Nagasaki, Japan; 3 The First Department of Oral and Maxillofacial Surgery, Graduate School of Dentistry, Osaka University, Osaka, Japan; 4 Department of Oral and Maxillofacial Surgery, School of Medicine, Nara Medical University, Kashihara, Japan; 5 Department of Dentistry and Oral Surgery, Shinshu University School of Medicine, Matsumoto, Japan; 6 Department of Oral and Maxillofacial Surgery, Keiyukai Sapporo Hospital, Sapporo, 003-0027 Japan; 7 Department of Oral and Maxillofacial Surgery, Kobe University Graduate School of Medicine, Kobe, Japan; 8 Department of Oral and Maxillofacial Surgery, Tokyo Dental College, Tokyo, Japan; 9 Oral and Maxillofacial Surgery, Department of Patho-biological Science, Graduate School of Dental Medicine, Hokkaido University, Sapporo, Hokkaido Japan; 10 Department of Oral and Maxillofacial Surgery, Faculty of Medicine, University of Miyazaki, Miyazaki, Japan; 11 Department of Dentistry Oral and Maxillofacial Surgery, Hyogo College of Medicine, Nishinomiya, Hyogo Japan; 12 Department of Dentistry, Oral and Maxillofacial Surgery, Jichii Medical University, Shimotsuke, Tochigi Japan; 13 Department of Oral and Maxillofacial Surgery, Hachioji Hospital, Tokai University, Tokyo, Japan; 14 Department of Dentistry and Oral Surgery, Iwaki Kyoritsu General Hospital, Iwaki, Fukushima Japan; 15 Department of Clinical Epidemiology and Biostatistics, Graduate School of Medicine, Osaka University, Osaka, Japan

## Abstract

**Background:**

This multicenter retrospective study aimed to determine whether elective neck dissection (END) can be performed for T1-2N0M0 tongue cancer.

**Methods:**

Patients with T1-2N0M0 tongue squamous cell carcinoma who received treatment between January 2000 and December 2012 were enrolled at 14 multicenter study sites. The 5-year overall survival (OS) and 5-year disease-specific survival (DSS) were compared between the propensity score-matched END and observation (OBS) groups.

**Results:**

The results showed that the OS rates among the 1234 enrolled patients were 85.5% in the END group and 90.2% in the OBS group (*P* = 0.182). The DSS rates were 87.0% in the END group and 94.3% in the OBS group (*P* = 0.003). Among the matched patients, the OS rates were 87.1% in the END group and 76.2% in the OBS group (*P* = 0.0051), and the respective DSS rates were 89.2% and 82.2% (*P* = 0.0335).

**Conclusion:**

This study showed that END is beneficial for T1-2N0M0 tongue cancer. However, END should be performed for patients with a tumor depth of 4–5 mm or more, which is the depth associated with a high rate of lymph node metastasis. The use of END should be carefully considered for both elderly and young patients.

The appropriate management for early-stage node-negative oral cancer remains a controversial issue. Previous studies have discussed whether elective neck dissection (END) should be performed for early-stage node-negative oral cancer.^1^^–^7 Although END may control occult lymph node metastasis, the procedure involves the risk of excessive surgical stress for the patient.

Furthermore, because these previous studies to determine whether END should be performed were commonly single-centered and the sample sizes were small, no definitive conclusion has been reached to date.^1^^–^7 Treatment of T1-2N0M0 oral cancer includes surgical excision according to the National Comprehensive Cancer Network (NCCN) guidelines. With regard to operative therapy, it is strongly recommended that END be performed when tumors are more than 4 mm in thickness.^8^ That is, the operative procedure cannot be selected solely on the basis of conventional clinical T stage. Rather, it is necessary also to consider tumor thickness.

We conducted a multicenter retrospective study to determine whether END should be performed for patients with T1-2N0M0 tongue cancer. Furthermore, although the study was retrospective, propensity scores were used, particularly to adjust, where possible, for background factors when differences in tumor depth between the two groups were expected.

## Patients and Methods

### Study Population

A multicenter study was conducted at 14 medical institutions involving patients with T1-2N0M0 tongue squamous cell carcinoma treated between January 2000 and December 2012. No patient had received preoperative treatment. Patients who received treatment (adjuvant therapy) after END were included.

All the patients underwent preoperative neck ultrasonography/computed tomography (CT) and/or magnetic resonance imaging (MRI)/chest radiology for diagnosis of T1-2N0M0. Tongue cancers were resected with a 1-cm margin. In principle, supraomohyoid neck dissection was performed as END.

Postoperative follow-up care included monthly follow-up examinations and neck ultrasonography plus CT, MRI, or both every 1–3 months for more than 2 years. The data collected included demographic information, age, sex, performance status (Eastern Cooperative Oncology Group), clinical T stage, END or observation (OBS) group, histologic grade, tumor depth (distance from a virtual line on the mucosa, between the normal mucosal membranes in the peripheral zone of the tumor measured on the pathologic specimen, to the deepest part of the tumor),^9^ and the year of operation. The distance from the virtual mucosal surface to the deeper part of the tumor, instead of tumor thickness, was used as a reference because it has been reported that the degree of tumor invasion is correlated with cervical lymph node metastasis.^9^^–^11

This study was approved by the research ethics committee of Tokai University School of Medicine (approval no. 15R-019). We obtained verbal informed consent from all the participants in the study.

### Statistical Analysis

To compare the baseline characteristics of the END and OBS patients, Chi-square (*χ*^2^) analysis was used for categorical variables and Wilcoxon rank-sum tests for continuous variables. To remove the effect of baseline imbalance in END efficacy, propensity score-matching was used to select a group of patients without neck dissection who had clinical and demographic characteristics similar to those of a group treated with END.

Propensity scores were computed using logistic regression with a variable indicating the presence or absence of END as an outcome, as a function of age, sex, performance status (Eastern Cooperative Oncology Group), clinical T stage, tumor depth, histologic grade, and year of operation.

Nonlinear restricted cubic splines were used to model nonlinearity of all continuous covariates together with interaction between age and tumor depth. Matching was performed based on the logit of the predicted probability of neck dissection estimated via multivariable logistic regression.

The balance of patient characteristics was compared by examining the balance of each variable between patients with and without END. The Kaplan–Meier product limit method was used to compute the cumulative proportion of patients without all-cause mortality among the matched population. These analyses were performed between both the original cohort and the propensity score-matched subjects.

To examine the observed differences between analyses using the original and matched cohorts, a multivariable Cox proportional hazard regression model was used to quantify the effect of END on survival, taking into account all covariates among the original cohort, together with the nonlinear effects of all continuous variables, as well as two- and three-way interactions between age, tumor depth, and neck dissection. Linear contrasts were computed from the multivariable Cox regression results to assess the effect of END for age- and tumor depth-specific populations of the original cohort.

To analyze the influence of death from other causes on all-cause mortality, we performed time-to-event analysis (Kaplan–Meier survival curves and log-rank tests), in which events were defined based only on disease-specific mortality. Patients who died of other causes were censored. In addition, competing risk analysis was performed to compute the cumulative incidence of disease-specific mortality in which patients who died of other causes were treated as competing risks together with use of Fine-Gray proportional hazards regression in a matched cohort.

All statistical inferences were made using two-sided tests at a 5% significance level, except in the interaction analyses. Because of the underpowered nature of interaction analyses, a two-sided significance level of 20% was used for all interactions.^12^

All statistical analyses were performed with R software, version 3.2.2 (F Foundation for Statistical Computing, Vienna, Austria) using the rms, Matching, and cmprisk packages.

## Results

### Original Cohort

The study recruited 1234 patients with T1-2N0M0 tongue cancer, including 131 in the END group and 1103 patients in the observation (OBS) groups. The patient demographic characteristics are listed in Table 1. The median follow-up period was 49 months (range 1–170 months). The rate of patients lost to contact was 3.2% (39 cases).

**Table 1:** Baseline and exercise characteristics according to neck dissection in the original cohort

	Neck dissection (*n* = 131) % (*n*)	Observation (*n* = 1103) % (*n*)	*P* value^a^
Age: years (range)	62 (20–92)	64 (18–96)	0.031
Sex			
Men	67.9 (89)	57.7 (636)	0.024
Women	32.1 (42)	42.3 (467)	
Performance status			
0	75.6 (99)	73.9 (815)	0.265
1	18.3 (24)	21.6 (238)	
2	6.1 (8)	3.5 (39)	
3	0 (0)	1.0 (11)	
Clinical T stage			
1	9.2 (12)	62.2 (686)	< 0.001
2	90.8 (119)	37.8 (417)	
Tumor depth (mm)	10.1 ± 5.2	3.7 ± 2.9	< 0.001
Histologic grade			
Well-differentiated	64.9 (85)	71.2 (785)	0.002
Moderately differentiated	27.5 (36)	24.5 (270)	
Poorly differentiated	7.6 (10)	2.3 (26)	
Carcinoma in situ	0 (0)	2.0 (22)	
Operation year	2007 (0.496 ± 3.787)	2007 (0.491 ± 3.608)	0.808

^a^Calculated using the Chi-square test for categorical variables and the Wilcoxon rank-sum test for continuous variables

The study groups differed significantly in terms of the following variables: sex, clinical T stage, tumor depth, and histologic grade. The fatal cases in the END group included death due to primary cancer for 4 patients, death due to neck cancer for 8 patients, death due to distant metastasis for 5 patients, and death due to other diseases for 2 patients. The fatal cases in the OBS group included death due to primary cancer for 11 patients, death due to neck cancer for 25 patients, death due to distant metastasis for 27 patients, and death due to other diseases for 44 patients.

In addition, salvage surgery was performed for 206 (94.1%) of 219 patients with neck metastases in the OBS group. To calculate *P* values, the Chi-square test was used for categorical variables and the Wilcoxon rank-sum test for continuous variables.

**Fig. 1:**
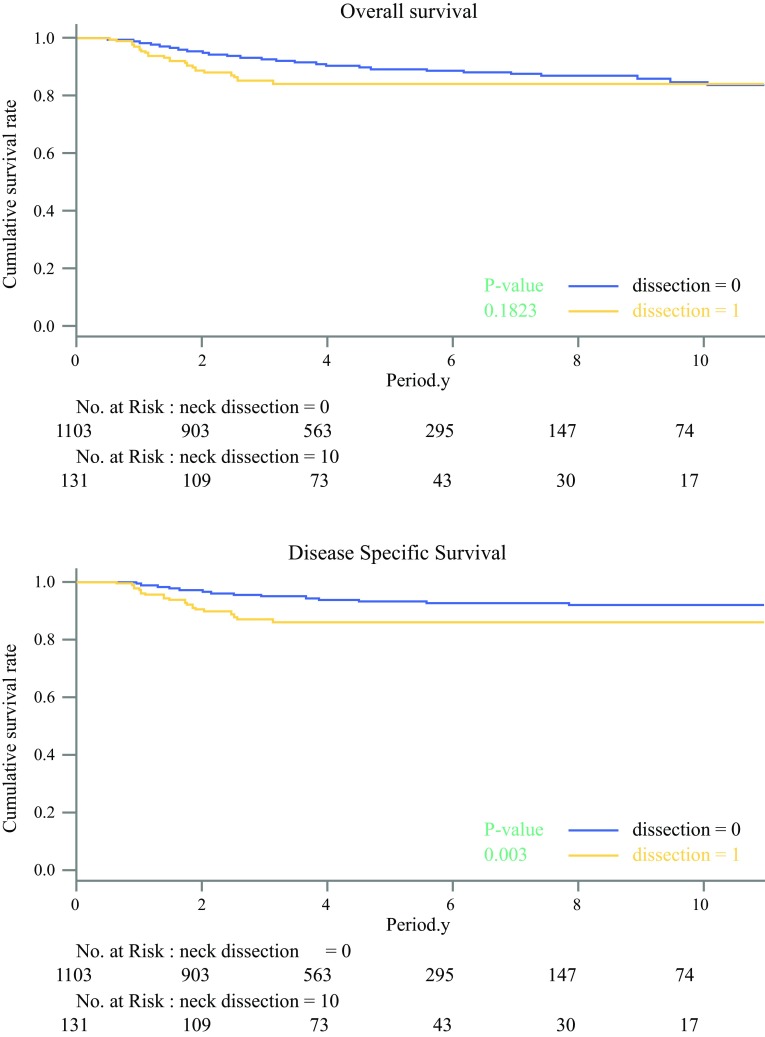
Kaplan–Meier curve relating overall survival (OS) and disease-specific survival (DSS) in the original cohort. The OS rates for the 1234 patients were 85.5% in the elective neck dissection (END) group and 90.2% in the observation (OBS) group (*P* = 0.182). The DSS rates were 87.0% in the END group and 94.3% in the OBS group (*P* = 0.003)

The overall survival (OS) rates for all the patients were 85.5% in the END group and 90.2% in the OBS group (*P* = 0.182). The disease-specific survival (DSS) rates were 87.0% in the END group and 94.3% in the OBS group (*P* = 0.003; Fig. 1).

### Propensity-Matched Patients

A total of 202 patients with T1-2N0M0 tongue cancer were matched: 101 patients in the END group and 100 patients in the OBS group. Table 2 shows the details for the 202 patients extracted by the propensity score method. The median follow-up period was 50 months (range 2–169 months). The significant differences between the groups were improved. To calculate *P* values, the Chi-square test was used for categorical variables and the Wilcoxon rank-sum test for continuous variables.

**Table 2:** Selected baseline and exercise characteristics according to neck dissection in propensity-matched patients

	Neck dissection (*n* = 101) % (*n*)	Observation (*n* = 101) % (*n*)	*P* value^a^
Age: years (range)	62 (20–92)	58 (20–92)	0.649
Sex			
Men	65.3 (66)	64.4 (65)	0.883
Women	34.7 (35)	35.6 (36)	
Performance status			
0	76.2 (77)	73.3 (74)	0.151
1	18.8 (19)	23.7 (24)	
2	5.0 (5)	1.0 (1)	
3	0 (0)	2.0 (2)	
Clinical T stage			
1	11.9 (12)	17.8 (18)	0.235
2	88.1 (89)	82.2 (83)	
Tumor depth (mm)	8.2 ± 3.5	8.4 ± 4.0	0.870
Histologic grade			
Well differentiated	69.3 (70)	64.4 (65)	0.584
Moderately differentiated	26.7 (27)	28.7 (29)	
Poorly differentiated	4.0 (4)	6.9 (7)	
Carcinoma in situ	0 (0)	0 (0)	
Operation year	2007 (0.317 ± 3.813)	2007 (0.158 ± 4.108)	0.938

^a^Calculated using the Chi-square test for categorical variables and the Wilcoxon rank-sum test for continuous variables

The postoperative treatments performed in the END group included radiation therapy for five patients, radiochemotherapy for two patients, and chemotherapy for one patient. The fatal cases in the END group included death due to primary cancer for two patients, death due to neck cancer for six patients, death due to distant metastasis for three patients, and death due to other diseases for two patients. The fatal cases in the OBS group included death due to primary cancer for two patients, death due to neck cancer for nine patients, death due to distant metastasis for seven patients, and death due to other diseases for six patients. In addition, salvage surgery was performed for 45 (93.8%) of the 48 patients with neck metastases in the OBS group.

**Fig. 2:**
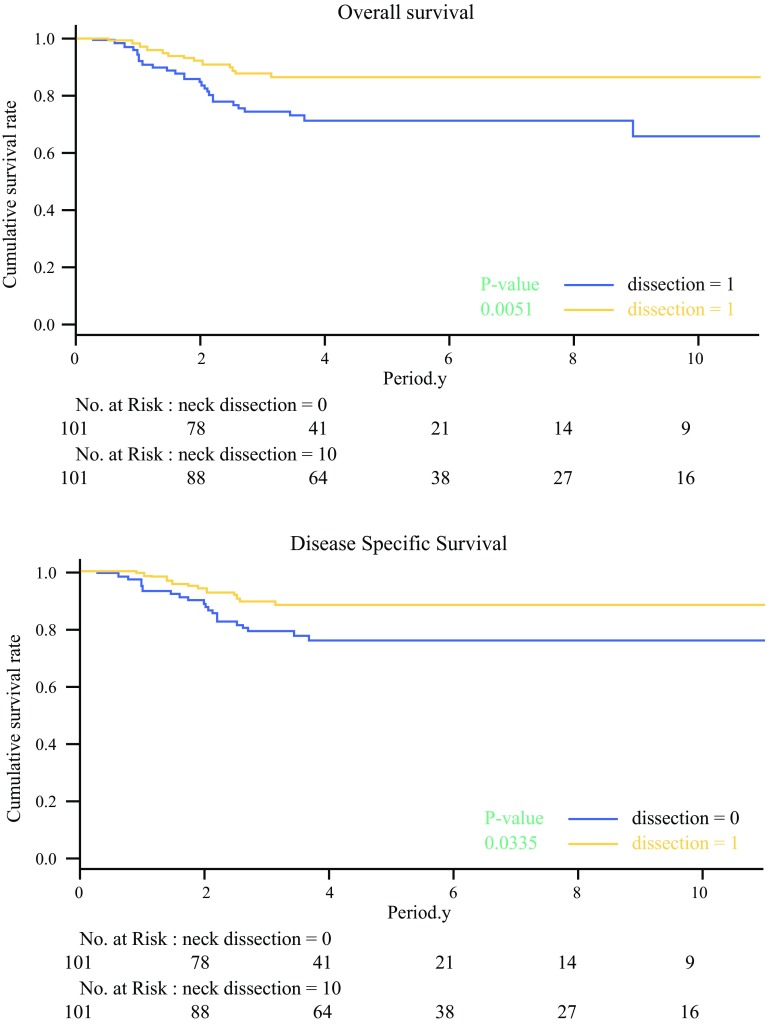
Kaplan–Meier curve relating overall survival (OS) and disease-specific survival (DSS) in the matched cohort. Among the matched patients, the OS rates were 87.1% in the elective neck dissection (END) group and 76.2% in the observation (OBS) group (*P* = 0.0051), and the DSS rates were respectively 89.1% and 82.2% (*P* = 0.0335)

The OS rates for the matched patients were 87.1% in the END group and 76.2% in the OBS group (*P* = 0.0051). The DSS rates were 89.1% in the END group and 82.2% in the OBS group (*P* = 0.0335; Fig. 2). The competing-risk analysis showed that the cumulative incidence of disease-specific death was significantly lower for the patients with END than for those without END (hazard ratio 0.47; 95% confidence interval [CI] 0.23–0.98; *P* = 0.043).

Occult lymph node metastasis, extracapsular spread (ECS), and number of metastatic lymph nodes were evaluated to compare pathologic lymph node status between the END and OBS groups. Occult lymph node metastases were observed in 23 (22.8%) of the 101 patients in the END group and 48 (47.5%) of the 101 patients in the OBS group (*P* < 0.001). Moreover, ECS was observed in 3 (13.0%) of the 23 patients in the END group and 18 (37.5%) of the 48 patients in the OBS group (*P* < 0.001). The mean number of metastatic lymph nodes was 1.8 in the END group and 2.3 in the OBS group (*P* = 0.022), showing poor prognostic significance in the OBS group.

### Multivariable Logistic Regression Analysis of Factors for Neck Dissection in the Original Cohort

Elective neck dissection was more likely to be performed for younger patients (*P* = 0.036) and those with deeper tumors (*P* < 0.001) (Table 3).

**Table 3:** Multivariable logistic regression analysis of factors in neck dissection among the original cohort

	Low	High	Effect	Lower 95% CI	Upper 95% CI	*P* value
Age	53	73	0.67	0.33	1.36	0.036
Tumor depth	2	6	14.60	5.6	38.1	< 0.001
Sex	Women	Men	1.53	0.95	2.48	0.080
Performance status	0	1	1.06	0.69	1.61	0.800
Clinical T stage	1	2	7.05	3.636	13.6	< 0.001
Histologic grade	Well	Moderately	1.02	0.608	1.70	0.761
Histologic grade	Well	Poorly	1.40	0.494	3.92	0.949
Histologic grade	Well	CIS	0.00	0	1.52 × 10^24^	0.532
Operation year	2005	2011	0.84	0.521	1.35	0.862

*CI* confidence-interval, *CIS* carcinoma in situ

### Cox Proportional Hazards Analysis of Time to Death in the Original Cohort

**Fig. 3:**
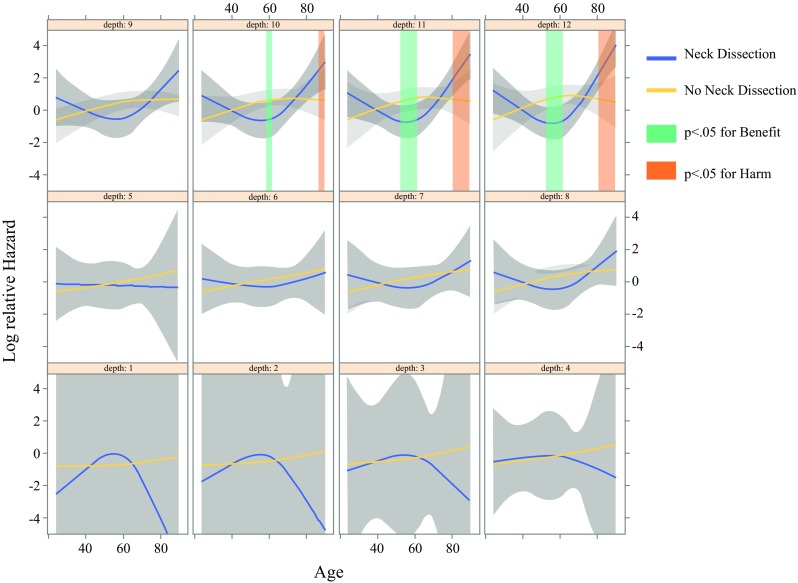
Cox proportional hazards analysis of time to death in the original cohort. Treatment was effective for the patients approximately 50–60 years of age with tumors deeper than 11 mm in the elective neck dissection (END) group compared with the observation (OBS) group (*P* < 0.05). Linear contrasts were computed from the multivariable Cox regression results to assess the effect of elective neck dissection **(**END**)** for the age- and tumor depth-specific population in the original cohort. The gray-shaded region represents the 95% confidence interval. The number of patients at risk shows the count of the following patients at the start time of each time point (*t*th) and does not include the patients who had an event or were censored by the *t* − 1th time point

The interaction between END, age, and tumor depth was significant (*P* = 0.057, Fig. 3). Treatment was effective for those approximately 50–60 years of age with tumors deeper than 11 mm in the END group compared with the OBS group (*P* < 0.05).

## Discussion

In 2015, D’Cruz et al.^13^ reported the results of a randomized controlled trial that examined the effect of END on patients with N0 oral cancer. The results showed that OS and DSS in the END group were significantly superior to those in the OBS group. Furthermore, the clinical benefits of END also were noted when the tumor was deeper than 3 mm. However, ultrasound assessment was not used for half of the patients during the post-surgery follow-up period, which reduced the reliability of these results.

In the study by D’Cruz et al.,^13^ 17.5% of patients in the OBS group had unresectable cancer with recurrence. In our research, only 3 (6.3 %) cases of unresectable cancer were detected in 48 patients. In addition, the results for tumor depth from the previous study showed confounding of cases, which affected the detection of jawbone and soft tissue cancers. However, these entities must be analyzed separately. Our analysis focused only on patients with tongue cancer (i.e., soft tissue cancer).

In 2011, Fasunla et al.^14^ reported the results from a meta-analysis of four randomized controlled trials that examined the effects of END. They reported that the disease-specific mortality rate was significantly superior in the END group to that in the OBS group. However, in these four trials, the number of patients was 40–70, which is a relatively small sample, and the follow-up period also was only about 3 years. Moreover, in the absence of significant differences in OS, the results are not very persuasive. Therefore, whether END should be performed or not remains inconclusive.

However, the NCCN guidelines strongly recommend performing END when tumors are more than 4 mm thick, and a clinical decision is necessary for tumors 2–4 mm in thickness.^8^ Several studies have reported that the number of lymph node metastases increases with a tumor depth of 4–5 mm.^9^^–^11 Therefore, we consider it necessary to reconfirm the effect of tumor depth on the prognosis of patients with tongue cancer.

Oral cancers such as cancers of the maxilla, mandible, or hard palate can cause bone invasion. In the current study, tumor thickness could not be compared with that in oral cancers occurring in other soft tissues. In addition, few cases of buccal mucosa/mouth floor cancers occurred in this study, so comparison is unrealistic. We therefore conducted a study restricted to T1-2N0M0 tongue cancer, which occurred in a relatively large number of patients, to determine whether END should be clinically performed or not for patients with tongue cancer. In addition, because the propensity score method was used in this study, it was possible to adjust and compare background factors, including tumor depth, in the two groups.

The results of the current study showed no evidence of a clinical benefit from END for all patients. However, for the patients matched using the propensity score method, OS (*P* = 0.0051) and DSS (*P* = 0.0335) in the END group were significantly superior to those in the OBS group. Among the matched patients, the possible reasons for the significantly superior survival rates observed in the END group include the possibility of having controlled micrometastases that cannot be detected in pathologic specimens stained with hematoxylin and eosin by performing END and by providing postoperative treatment at an appropriate time based on the results.^15^^–^19 In addition, cervical lymph node metastasis,^20^^,^^21^ ECS,^20^^,^^22^ and number of metastases 20^,^^21^ are important risk factors in oral cancer. In the current study, the rate of occult lymph node metastasis, the ECS rate, and the mean number of metastases were significantly higher in the OBS group. Then why did the effect of END change after propensity score-matching? To clarify this issue, Cox proportional hazards analysis of time to death in the original cohort was performed for nonlinear effects of all continuous variables as well as two- and three-way interactions between age, tumor depth, and END.

As described in the NCCN guidelines also, tumor depth is an important factor in the prognosis of oral cavity cancer. In addition, multivariable logistic regression analysis of the neck dissection in the original cohort identified age as a significant predictor of cancer risk. Thus, these two factors (tumor depth and age) were selected.^8^

The analysis showed interactions between age, tumor depth, and END (*P* = 0.0565). In this study, END was performed for younger patients (*P* = 0.0366) and for those with deeper tumors (*P* < 0.0001). We hypothesized that by extracting these patients, the effect of END would become significant among the propensity score-matched subjects. Furthermore, when the nonlinear effect was verified in detail, a significant survival benefit of END was observed for the patients approximately 50–60 years of age with tumors deeper than 11 mm (*P* < 0.05). However, this likely was influenced by the small sample size, which necessarily affected whether statistically significant differences could be detected. Considering clinical practice, we concluded that END should be performed for patients 40–60 years of age with tumors deeper than 4–5 mm.

These results conflict with the opinion held by D’Cruz et al.,^13^ that END should be applied to tumors with a depth of 3 mm or more. Furthermore, we should consider the burden that END puts on patients. Specifically, END may lead to shoulder syndrome, dysphagia, and reduced quality of life. As shown by our results, the decision to perform END should be made with consideration of both patient age and tumor depth.

Based on the results of this study, END is beneficial for T1-2N0M0 tongue cancer. However, END should be performed only for tumors deeper than 4–5 mm, the depth at which conventional lymph node metastases commonly occur, and the application of END should be considered carefully for young and elderly patients.
